# Observational study of adult respiratory infections in primary care clinics in Myanmar: understanding the burden of melioidosis, tuberculosis and other infections not covered by empirical treatment regimes

**DOI:** 10.1093/trstmh/trab024

**Published:** 2021-03-03

**Authors:** Clare E Warrell, Aung Pyae Phyo, Mo Mo Win, Alistair R D McLean, Wanitda Watthanaworawit, Myo Maung Maung Swe, Kyaw Soe, Htet Naing Lin, Yee Yee Aung, Chitmin Ko Ko, Cho Zin Waing, Kaung San Linn, Yadanar Phoo Wai Aung, Ne Myo Aung, Ni Ni Tun, David A B Dance, Frank M Smithuis, Elizabeth A Ashley

**Affiliations:** Myanmar Oxford Clinical Research Unit, Yangon, Myanmar; Medical Action Myanmar, Yangon, Myanmar; Myanmar Oxford Clinical Research Unit, Yangon, Myanmar; Department of Medical Research, Myanmar; Myanmar Oxford Clinical Research Unit, Yangon, Myanmar; Centre for Tropical Medicine and Global Health, Nuffield Department of Medicine, University of Oxford, Oxford, UK; Shoklo Malaria Research Unit, Mahidol-Oxford Tropical Medicine Research Unit, Faculty of Tropical Medicine, Mahidol University, Mae Sot, Thailand; Myanmar Oxford Clinical Research Unit, Yangon, Myanmar; Myanmar Oxford Clinical Research Unit, Yangon, Myanmar; Myanmar Oxford Clinical Research Unit, Yangon, Myanmar; Medical Action Myanmar, Yangon, Myanmar; Department of Medical Research, Myanmar; Medical Action Myanmar, Yangon, Myanmar; Medical Action Myanmar, Yangon, Myanmar; Medical Action Myanmar, Yangon, Myanmar; Medical Action Myanmar, Yangon, Myanmar; Department of Medicine, Insein General Hospital, Min Gyi Road, Insein Township, Yangon, Myanmar; Department of Medicine, University of Medicine 2, Khaymar Thi Road, North Okkalapa Township, Yangon, Myanmar; Myanmar Oxford Clinical Research Unit, Yangon, Myanmar; Medical Action Myanmar, Yangon, Myanmar; Centre for Tropical Medicine and Global Health, Nuffield Department of Medicine, University of Oxford, Oxford, UK; Lao-Oxford-Mahosot Hospital-Wellcome Trust Research Unit, Microbiology Laboratory, Mahosot Hospital, Vientiane, Lao People's Democratic Republic; Faculty of Infectious and Tropical Diseases, London School of Hygiene and Tropical Medicine, London, UK; Myanmar Oxford Clinical Research Unit, Yangon, Myanmar; Medical Action Myanmar, Yangon, Myanmar; Myanmar Oxford Clinical Research Unit, Yangon, Myanmar; Lao-Oxford-Mahosot Hospital-Wellcome Trust Research Unit, Microbiology Laboratory, Mahosot Hospital, Vientiane, Lao People's Democratic Republic; Faculty of Infectious and Tropical Diseases, London School of Hygiene and Tropical Medicine, London, UK

**Keywords:** *Burkholderia pseudomallei*, influenza, melioidosis, Myanmar, respiratory infection, tuberculosis

## Abstract

**Background:**

Lower respiratory infections constitute a major disease burden worldwide. Treatment is usually empiric and targeted towards typical bacterial pathogens. Understanding the prevalence of pathogens not covered by empirical treatment is important to improve diagnostic and treatment algorithms.

**Methods:**

A prospective observational study in peri-urban communities of Yangon, Myanmar was conducted between July 2018 and April 2019. Sputum specimens of 299 adults presenting with fever and productive cough were tested for *Mycobacterium tuberculosis* (microscopy and GeneXpert MTB/RIF [*Mycobacterium tuberculosis*/resistance to rifampicin]) and *Burkholderia pseudomallei* (Active Melioidosis Detect Lateral Flow Assay and culture). Nasopharyngeal swabs underwent respiratory virus (influenza A, B, respiratory syncytial virus) polymerase chain reaction testing.

**Results:**

Among 299 patients, 32% (95% confidence interval [CI] 26 to 37) were diagnosed with tuberculosis (TB), including 9 rifampicin-resistant cases. TB patients presented with a longer duration of fever (median 14 d) and productive cough (median 30 d) than non-TB patients (median fever duration 6 d, cough 7 d). One case of melioidosis pneumonia was detected by rapid test and confirmed by culture. Respiratory viruses were detected in 16% (95% CI 12 to 21) of patients.

**Conclusions:**

TB was very common in this population, suggesting that microscopy and GeneXpert MTB/RIF on all sputum samples should be routinely included in diagnostic algorithms for fever and cough. Melioidosis was uncommon in this population.

## Introduction

Lower respiratory tract infections (LRTIs) were identified as the leading infectious cause of death worldwide and the fifth overall biggest killer in the 2015 Global Burden of Disease study.^1^ Conventional empirical therapy covering common community-acquired bacterial pathogens is widely used in healthcare settings for suspected bacterial LRTIs. Understanding the local prevalence of pathogens not susceptible to first-line empirical treatment is important for structuring population-specific syndromic treatment guidelines.

Melioidosis was first discovered in Yangon (formerly Rangoon), Myanmar in 1911.^2,3^ It is now an emerging tropical infection causing an estimated 165 000 cases and 89 000 deaths worldwide each year, exceeding many recognised neglected tropical diseases, but it is greatly underdiagnosed.^4^ Pneumonia, secondary to either *Burkholderia pseudomallei* inhalation or bacteraemic spread to the lung, is the most common presentation (51%).^5^ Annually the majority of reported cases present in the rainy season (75% in Thailand) and up to 80% of patients have at least one recognised risk factor, including diabetes, alcohol dependence, glucocorticoid therapy, chronic obstructive pulmonary disease, chronic renal disease and cancer.^6^ Human immunodeficiency virus (HIV) infection does not appear to increase the risk of melioidosis.^7^ Recent soil sampling surveys have demonstrated the presence of *B. pseudomallei* in central Myanmar, including Yangon,^8,9^ yet there have been few published clinical cases in Myanmar since 1942.^10,11^ Difficulties diagnosing *B. pseudomallei* in resource-poor endemic regions include lack of physician awareness of the disease, clinical and radiological mimicry of other bacterial infections, especially tuberculosis (TB),^12^ and limited access to specialist laboratory facilities and expertise required for diagnosis.^11^ Development of *B. pseudomallei* rapid tests could expedite diagnosis and facilitate identifying communities where melioidosis is currently an undescribed source of LRTI and sepsis.

Myanmar is classified as a high-burden country for TB. According to the Global Tuberculosis Report 2020, in 2019 the incidence of TB was 322/100 000 population (uncertainty interval 212–454), the HIV prevalence among TB incident cases was 7.8% and the mortality incidence in HIV-uninfected and HIV-infected individuals with TB was 36 (95% confidence interval [CI] 21 to 54) and 5.8 (95% CI 3.8 to 8.1), respectively. The estimated percentage with multidrug-resistant (MDR) or rifampicin-resistant TB among new cases was 4.9% (95% CI 4.7 to 5.1) and among previously treated cases was 18% (95% CI 17 to 19).^13^

The primary aims of this study were to identify the proportion of cases of LRTI caused by *B. pseudomallei* during the wet season among adults attending outpatient clinics in Yangon and to evaluate the performance of a new rapid test, the Active Melioidosis Detect Lateral Flow Assay (AMD-LFA; InBios, Seattle, WA, USA) on sputum compared with culture (the gold standard). A secondary objective was to identify leading alternative causes of LRTI that would not be effectively treated by the usual empirical treatment (oral amoxicillin)^14^ for LRTI in this population (i.e. TB and respiratory viruses).

## Methods

### Study area and population

This was a prospective observational study of patients attending two outpatient clinics in Hlaingtharyar and Shwepyithar townships, both large, crowded slums of Yangon, built on paddy fields, with an estimated combined population of 1.3 million. Inhabitants of these townships are generally poor, working in factories or construction and to a lesser extent in farming. Unemployment rates are high.

Medical Action Myanmar (MAM), a medical aid organisation, runs clinics providing free healthcare in these townships 7 d a week. These clinics provide general healthcare and specialist services in HIV management. Chest infections are among the most common presentations in adults at the clinic. Risk factors for both TB and melioidosis (e.g. diabetes, alcohol abuse^15,16^ and renal impairment^17^) are common in the region. The inclusion criteria were consenting patients ≥18 y of age reporting fever or having a measured axillary temperature ≥37.5°C and a productive cough. Patients unable to provide sputum specimens were excluded.

Data collected included demographic variables and self-reported or previously documented risk factors (age, gender, occupation, smoking history, diabetes status, alcohol dependence [defined as the need to imbibe alcohol daily to suppress physical symptoms of withdrawal], smoking and betel chewing status, chronic lung disease, renal disease, drug history including current steroid use, HIV status, record of any recent injury, near drowning and occupational contact with soil) and symptoms (reported fever, productive cough, haemoptysis, rigors, weight loss, night sweats, shortness of breath and chest pain). Vital signs, weight and physical examination findings were recorded on case report forms. Patients were managed in accordance with the current clinic guidelines.^14^ Empirical treatment with amoxicillin or azithromycin was given to patients suspected of having bacterial infection while awaiting investigation results with 1-week follow-up.

### Laboratory investigations

#### Sputum

Specimens were collected for acid-fast bacilli (AFB) microscopy, GeneXpert MTB/RIF (*Mycobacterium tuberculosis*/resistance to rifampicin; Cepheid, Sunnyvale, CA, USA), AMD-LFA rapid diagnostic test (InBios) and selective *B. pseudomallei* culture (MacConkey and Ashdown agar and selective enrichment broth). Ashdown plates were incubated for 4 d. Enrichment broths were incubated for 48 h at 37°C then subcultured on Ashdown agar for 4 d. Gram stain for morphology of bacteria, API 20NE (bioMérieux, Marcy-l’Étoile, France) and latex agglutination were used to confirm the identity of *B. pseudomallei*.^18^ Positive isolates were sent to the Menzies School of Health Research, Darwin, NT, Australia for confirmation of identity by specific polymerase chain reaction (PCR) and whole genome sequencing (WGS).

#### Nasopharyngeal swabs

Specimens were frozen at −80°C and underwent respiratory virus real-time PCR assays for detection of respiratory syncytial virus (RSV), influenza A (including H1N1 typing) and influenza B at the Shoklo Malaria Research Unit in Bangkok, Thailand.

#### Blood

Patients had blood tests for renal function (creatinine and urea), full blood count (haemoglobin, platelets, white cell count), random blood glucose, C-reactive protein (CRP) and HIV. Blood cultures were taken from patients who were identified as septic with a quick sepsis-related organ failure assessment (qSOFA) score ≥2 and sent to the National Health Laboratory, Yangon, where they underwent automated blood culture with identification and antibiotic susceptibility testing by VITEK 2 (bioMérieux).

#### Imaging

Chest radiographs were carried out by local radiology services and the reports were documented and plain films were reviewed by the clinicians to guide management.

### Sample size calculation and statistical analysis

The formula used to calculate the sample size was

\begin{eqnarray*} &&\text{sample size required = }\rm {\left [(1.96^{2})* \rm{prevalence} \right.}\\ &&*\left.(1 - {\rm{prevalence}}) \right ]/({\rm{precision}^{2}}). \end{eqnarray*}

We considered what prevalence of melioidosis would be required to encourage the introduction of a diagnostic test as routine and we estimated that a sample size of 283 patients, assuming a target population prevalence of *B. pseudomallei* of 10%, would provide precision of 3.5% with 95% confidence. This number was rounded up to 300.

For comparisons of continuous variables between TB-positive and TB-negative patients, two-sample Wilcoxon rank sum (Mann–Whitney) tests were used. The 95% CIs for prevalences were calculated using exact binomial (Clopper–Pearson) CIs. Statistical analyses were performed using Stata 14.2 (StataCorp, College Station, TX, USA).

## Results

The study took place between 9 July 2018 and 4 April 2019. The baseline characteristics of the 299 patients meeting the inclusion criteria are presented in Table 1. A total of 67% of study subjects were male, the median age was 42 y (25th–75th percentile 25–50) and the median body mass index was 18.8 (25th–75th percentile 17.3–21.8).

**Table 1.** Clinical and demographic factors of the study population

Demographic factors (N=299)	Values
Age (years)	42 (29–50)
Male, n (%)	200 (67)
Weight (kg)	48 (43–56)
Height (cm)^a^	160 (152–165)
Occupation (N=299), n (%)	
Farmer	25 (8.4)
Manual worker	69 (23)
Housewife	29 (9.7)
Student	3 (1)
Construction worker	34 (11)
Unemployed	46 (15)
Other	93 (31)
Presenting history (N=299)	
Fever duration (days)^a^	7 (3–18)
Productive cough duration (days)^a^	10 (5–30)
Weight loss, n (%)	161 (54)
Night sweats, n (%)	136 (46)
Chest pain, n (%)	146 (49)
Difficulty breathing^a^, n (%)	164 (55)
Rigors^a^, n (%)	72 (24)
Myalgia, n (%)	186 (62)
Lethargy^a^, n (%)	193 (65)
Concurrent medical issues (N=290), n (%)	
Diabetes mellitus	14 (4.8)
HIV	10 (3.4)
Chronic lung disease	12 (4.1)
Chronic kidney disease	5 (1.7)
Cancer	4 (1.4)
Previous TB^b^	63 (21)
Social history (N=299), n (%)	
Alcohol dependence	49 (17)
Betel chewer	151 (52)
Smoker or ex-smoker	127 (44)
Steroid use	24 (8.3)
Intravenous drug user	3 (1)
Medication (N=299), n (%)	
Prior antibiotic treatment for this infection	55 (18)
Prior treatment with traditional medicine	32 (11)
Observations (N=299)	
Axillary temperature (°C)^a^	37.0 (36.5–37.7)
Respiratory rate (breaths/min)^a^	20 (18–20)
Oxygen saturation (% room air)	97 (96–98)
Heart rate (beats/min)	92 (84–108)
Systolic blood pressure(mmHg)^a^	110 (100–120)
Investigations (N=299)	
Haemoglobin (g/dl)	13.0 (11.7–14.3)
White cell count (×10^9^/L)	9.8 (7.9–12.6)
Creatinine (mg/dL)^a^	1.00 (0.85–1.10)
CRP (mg/L)^a^	29 (9–75)

Data are presented as median (25th–75th percentile) unless stated otherwise.

^a^n<299 due to absent data.

^b^n=298.

In the month prior to presentation, 34% (103/298) had spent time in paddy fields, 3.0% (9/298) reported near-drowning experiences and 14% (43/298) had had skin-breaching injuries. Other factors known to be associated with melioidosis were alcohol dependence in 17% (49/290) and current steroid use in 8.3% (24/290). At presentation 4.8% (14/290) of patients had a known diagnosis of diabetes and one new diagnosis was made during the study. Prior to presentation, 18% (55/298) of patients had already taken antibiotics, either obtained over the counter or in other health centres (amoxicillin 12, co-amoxiclav 11, flucloxacillin 1, azithromycin 1, ciprofloxacin 1, metronidazole 1, cefixime 1, unknown 27).

Chest radiographs were abnormal in 73% (166/226) of patients. Local radiologist–reported radiographs were 81% sensitive and 79% specific for the detection of TB, with an 89% negative predictive value. Four patients with films locally reported as having ‘no abnormalities detected’ tested positive on laboratory tests for *M. tuberculosis*; one was HIV positive.

*B. pseudomallei* was detected in one patient by AMD-LFA and culture (see Box 1). Specific PCR and WGS confirmed this was a *B. pseudomallei* strain of a novel multilocus sequence typing type (ST 1765) (https://pubmlst.org/bpseudomallei/).^10^ Susceptibility testing of the isolate confirmed that it was resistant to gentamicin and colistin and susceptible to co-amoxiclav, co-trimoxazole, ceftazidime and meropenem.

Box 1. **Confirmed melioidosis case**

A 38-year-old presented in rainy season with a 2-week history of cough, fever, weight loss and lethargy. He was a manual worker with regular exposure to paddy fields in the previous month. He was a betel chewer and cigarette smoker, with no previous medical history. On examination his temperature was 37.0°C, respiratory rate 29 breaths/min, oxygen saturation 92% on room air, heart rate 92 beats/min and blood pressure 120/80 mmHg. He had already sought medical advice the previous week elsewhere and had not responded to a short course of co-amoxiclav and cefixime. On examination he was expectorating yellow sputum with no abnormal chest findings or lymphadenopathy. His investigations showed a random blood sugar of 131 mg/dL, haemoglobin 12 g/dL, platelets 560, Urea 9 mg/dL and CRP 44 mg/L. His chest radiographs showed a left-sided large lung abscess in the middle zone. Negative results included HIV, sputum AFB and GeneXpert. *B. pseudomallei* grew on Ashdown agar plates. He was transferred to a local hospital where he was treated with 2 weeks of intravenous ceftazidime 2 g every 8 h. He was subsequently followed up in clinic and switched to co-trimoxazole 960 mg twice a day for 12 weeks. On completion of therapy he had clinically recovered and a repeat chest radiograph confirmed complete resolution of infection.

Evidence of TB was found in 32% of the study population (95% CI 26 to 37). Seventy patients were positive by both microscopy and GeneXpert MTB/RIF, 20 by GeneXpert MTB/RIF alone and 5 by microscopy alone. Rifampicin resistance was identified in nine of these cases.

**Figure 1.**
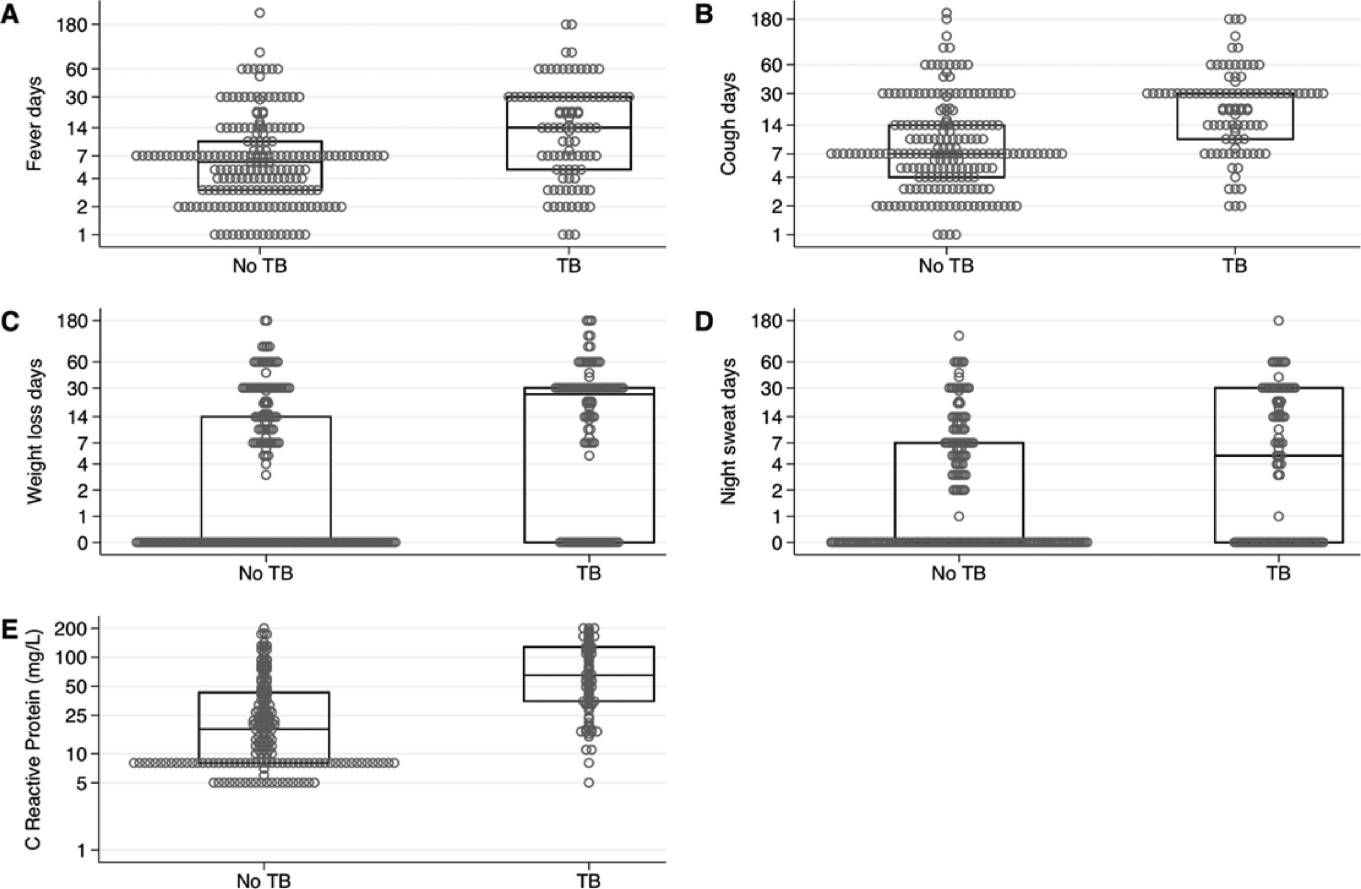
Comparison of symptom duration and CRP in patients with and without TB. (**A**) Fever duration was a median of 15 d in TB patients compared with 6 d in non-TB patients (p<0.0001). (**B**) Productive cough duration was 30 d in TB patients versus 7 d in non-TB patients (p<0.0001). (**C**) Weight loss. (**D**) Night sweats. (**E**) Serum CRP (mg/L) measurements. Dots denote each patient, boxes denote 25th, 50th and 75th percentiles. Note that days and CRP are presented on a log scale and for panels (C) and (D), zero values are displayed at 0.5 for clarity.

Figure 1 shows the presence and duration of cardinal symptoms of TB (fever, productive cough, weight loss and night sweats) and CRP at presentation in patients with and without TB. Previous treatment for TB was reported in 21% (63/298). Symptom duration was longer in TB-positive versus negative patients. The median fever duration was 6 d (25th–75th percentile 3–10) in non-TB patients and 15 d (25th–75th percentile 5–30) in TB patients (p<0.0001) and productive cough had been present for a median of 7 d (25th–75th percentile 4–14) in non-TB patients and 30 d (25th–75th percentile 10–30) in TB patients (p<0.0001). More than a quarter of patients (29%) reported known contact with at least one relative or friend with TB. The median CRP of the non-TB patients was 18 mg/L (25th–75th percentile 8–43) compared with 65 mg/L (25th–75th percentile 35–128) in TB patients (p<0.0001). Among the diabetic patients, 40% (6/15) presenting with fever and cough tested positive for TB.

**Figure 2.**
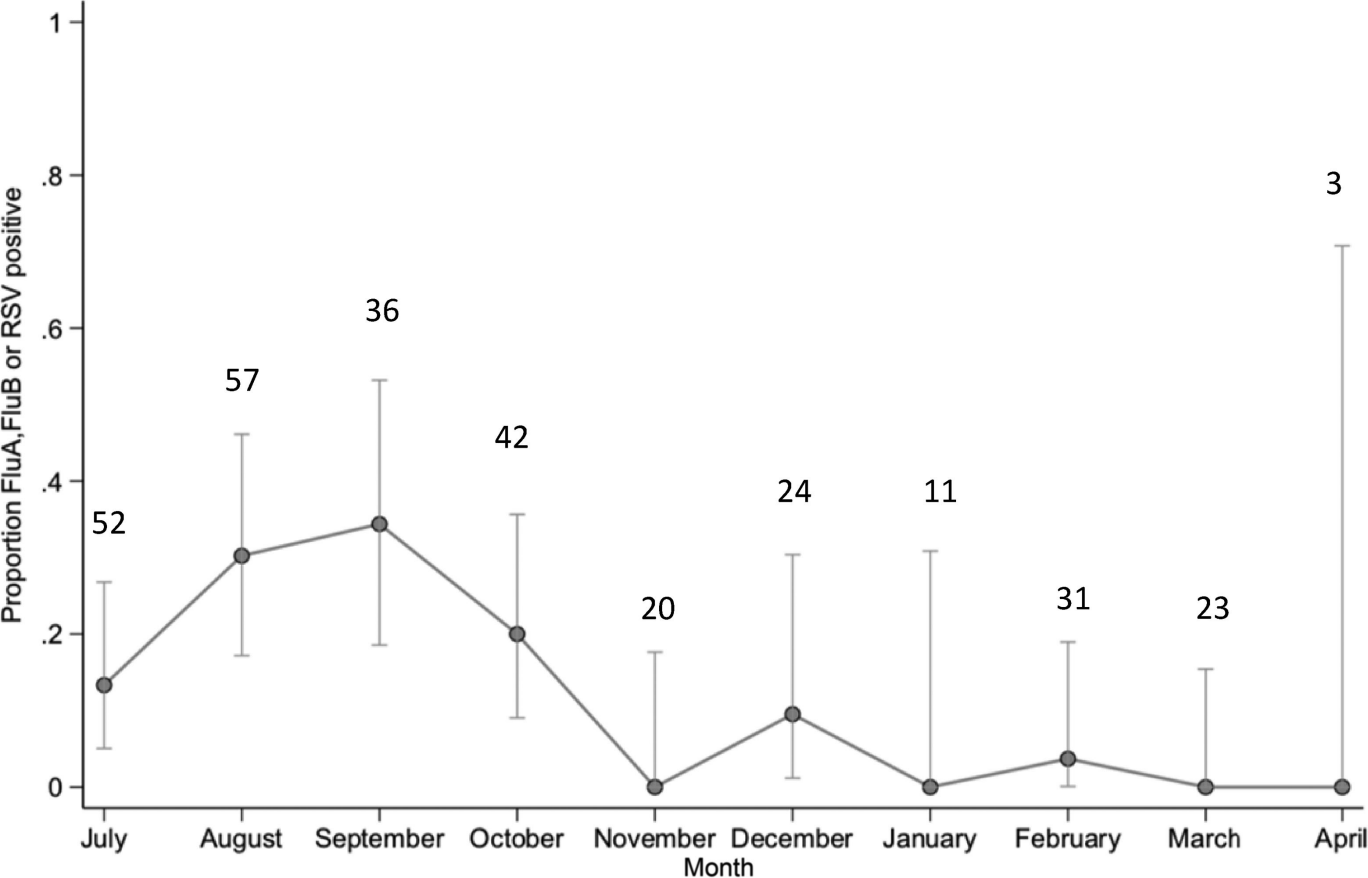
Proportion of patients with respiratory virus (influenza A, influenza B and RSV) positive by month in 2018–2019 (n=299). Circles denote observed proportions, capped vertical bars denote the 95% CIs. Number of patients contributing to each month are the numbers over each data point.

Using the respiratory virus real-time PCR assays, influenza A was detected in 5.2% (95% CI 3.1 to 8.5) of nasopharyngeal swabs, influenza B in 6.6% (95% CI 4.2 to 10) and RSV in 4.4% (95% CI 2.6 to 7.6) (Table 2). Figure 2 shows viral diagnoses were made predominantly in the wet season months of July–October.

**Table 2.** Potential pathogens identified from sputum or nasopharyngeal swabs

Bacterial culture (non-mycobacterial) (N=299)	n	%	95% CI
*Burkholderia pseudomallei*	1	0.3	0 to 18
*Burkholderia cepacia*^a^	1	0.3	0 to 18
TB (sputum smear and/or GeneXpert MTB/RIF positive; N=298)			
TB	95	32	26 to 37
Rifampicin-resistant TB	9	3	1.6 to 5.6
Viruses (N=271)			
Influenza A (all)	14	5.2	3.1 to 8.5
Influenza A (H1N1)	0	0	0 to 0
Influenza B	18	6.6	4.2 to 10
RSV	12	4.4	2.6 to 7.6
No pathogen detected	88	29	24 to 35

^a^Likely to represent colonisation.

A potential bacterial or viral pathogen was detected in 46% (95% CI 41 to 52) of cases (Table 2). Three patients had TB and virus co-infection (one influenza A, one influenza B and one RSV). Blood cultures were sent in two patients but were sterile.

At presentation, 3.4% (10/290) of patients had known HIV infection and 19 (6.6%) people refused point-of-care HIV investigation at pre-test counselling. During the study, 19 (6.5%) new cases of HIV infection were diagnosed. TB co-infection was diagnosed in 9 (31%) HIV-positive patients.

## Discussion

Although this study was originally designed to investigate the prevalence of melioidosis in patients presenting with LRTIs in the MAM clinics, it has confirmed the importance of *M. tuberculosis* (32%) as a leading cause of fever and productive cough in this population. Myanmar is among the 30 countries worldwide with the highest burden of TB.^13^ Results from the 2018 national TB prevalence survey for the Yangon region found 108 of 3325 (3.2%) eligible adult participants tested had bacteriologically confirmed active TB, a prevalence which is 10-fold lower than in this study.^19^ In the same survey, 4/108 (3.7%) individuals in Yangon were diagnosed with rifampicin-resistant TB, which is highly suggestive of MDR-TB. In 2016, 54% of 2544 MDR-TB cases started on second-line drugs nationally were in Yangon. In 2019 the estimated percentage of new cases nationally with MDR or rifampicin-resistant TB was 4.9% (95% CI 4.7 to 5.1) and HIV prevalence among TB-incident cases in Myanmar was 7.8%.^13^ Rifampicin resistance was present in 9 of 70 patients (13%) diagnosed with TB in our study and 9 (13%) patients were co-infected with HIV. Around 17% (49/290) of patients reported alcohol dependence. In a published survey between 2013 and 2014 of 1486 adults residing in urban and rural areas of the Yangon region, age-standardised prevalence of heavy drinking (defined as >40 g alcohol/d for men and >20 g for women) was <5%.^20^ The catchment area served by the study clinics is home to one of the most deprived populations in Myanmar, many of whom moved to Yangon from the Delta region after Cyclone Nargis in 2008. People are living in poverty in overcrowded conditions. It is probable that rates of TB transmission are particularly high in this area, which warrants further investigation. In addition, 55 (18%) patients presented having already taken a course of antibiotics, which is very common in Southeast Asian countries where antibiotics are readily available over the counter. Hence only those not responding to this initial empirical treatment would continue to seek medical advice and are more likely to have a more complex infection.

The high proportion of TB diagnosed in this study, and the greater ascertainment with molecular as opposed to microscopic testing, suggests that screening all patients with a productive cough and fever with routine GeneXpert MTB/RIF testing should be considered in local diagnostic protocols.

TB was presumed to be the diagnosis in five patients (age range 19–53 y, median 39 y) who were microscopy positive and GeneXpert MTB/RIF negative, although non-tuberculous mycobacterial (NTM) infection is an alternative explanation, as discussed by Phyu et al.^21^

Baseline CRP was significantly higher in TB patients than non-TB patients (p<0.0001) (see Figure 1), but whether this supports introduction of routine CRP testing as a point-of-care screening or diagnostic tool for differentiating TB in this setting requires a better-powered study. The value of CRP as a marker of mycobacterial infection in European patients was dependent on ethnicity, the species of mycobacterium and the site of disease.^22^ In view of the high proportion of this population found to have TB, active case finding may be better than passive case detection in controlling the rates of infection, as demonstrated by Marks et al.^23^ in Vietnam.

The proportion of HIV-positive patients with TB (31% [9/29]) was comparable to that in HIV-negative patients (32% [86/270]). The diagnosis of 18 new HIV cases during this study emphasises the importance of opportunistic testing in patients presenting to healthcare centres with infections in areas of high prevalence. The high proportion of new HIV infections is not representative of the population as a whole, given that this clinic offers HIV specialist services and may consequently attract a higher-risk population.

Studies in Thailand and Cambodia have identified melioidosis as an important TB mimic that should be considered in patients testing negative for TB but presenting with similar respiratory infection.^12,24^*B. pseudomallei* remains an important differential for clinicians to consider in patients in melioidosis-endemic countries. This study confirms that melioidosis still exists in Yangon and should be considered as a cause of LRTIs, although it was uncommon in this study population. The AMD-LFA, which detects *B. pseudomallei* 6-deoxyheptan capsular polysaccharide antigen, has been specifically developed for resource-poor, melioidosis-endemic settings where *B. pseudomallei* culture is frequently not available.^25^ It has been evaluated on blood culture broths in Cambodia and Laos^26^ and directly on clinical samples in Australia,^27^ Laos,^28,29^ Thailand^30^ and India.^31^ Shaw et al.^31^ demonstrated an overall sensitivity of 86% (CI 75 to 93) and specificity of 94% (CI 88 to 97) with a positive predictive value of 86% (CI 76 to 92) compared with culture on multiple types of specimens. The estimated bacillary load of *B. pseudomallei* in sputum and pus is 1.1×10^5^ CFU/mL, higher than other clinical samples (e.g. blood), making them promising specimen types for AMD-LFA testing.^26,27,32^ In the respiratory samples (n=45) tested by Shaw et al.,^31^ the AMD-LFA detected all five culture-positive samples. In Laos, a study by Woods et al.^29^ found 100% specificity and 33% sensitivity on sputum (n=20), while a further study in Laos^28^ demonstrated a specificity of 100% and sensitivity of 80% in sputum specimens (n=28). Reduced time to diagnosis with this novel test has previously been demonstrated in Laos.^28^ In our patient, the diagnosis of melioidosis was expedited by the rapid diagnostic test. The AMD-LFA was accurate and fast, providing a positive result within 15 min of testing the sputum, 1 week before the diagnosis was confirmed by culture. This enabled rapid commencement of appropriate treatment (ceftazidime) in a patient who would otherwise have been empirically commenced on inappropriate antibiotics, with potentially fatal consequences. At this stage, the likely market price of the AMD-LFA is unknown and so we cannot comment on the costs and benefits of its routine use in this population, although targeted use in specific risk groups and patients with severe pneumonia and sepsis is likely to be most cost effective.

Contributory factors to the low prevalence of *B. pseudomallei*–associated LRTIs in this population include logistical delays leading to recruitment to the study starting 2 months into the rainy season. A majority of the patients were recruited in the lower-risk risk dry months. More acutely unwell patients may have presented directly to hospital rather than to community outpatient clinics.

Diabetes was a risk factor for TB in this study, with 40% (6/15) of diabetics presenting with a productive cough testing positive. Diabetes mellitus is among the top 10 causes of death in Myanmar and is thought to be largely undiagnosed.^33^ Immunosuppression caused by glycaemic dysregulation predisposes the patient to a wide range of infections. A systematic review of 2.3 million patients with active TB globally estimated a pooled international prevalence of diabetes of 15.3%. The regional diabetes prevalence in Southeast Asia is 19%.^34^ This association supports screening patients with TB for diabetes at diagnosis and vice versa. Diabetes is also the leading risk factor for melioidosis, increasing the risk of the infection 12-fold.^35^

Real-time respiratory virus PCR testing showed seasonal influenza peaks from June to October. The majority of the cases were influenza B, which developed after an initial wave of influenza A, and was followed by RSV infections. This is in keeping with published Myanmar surveillance records.^36^

## Conclusions

This study demonstrates an unexpectedly high proportion of infections that would not be expected to respond to the usual empirical antibiotic treatment regimens, particularly TB, in this study population in Yangon. Screening all patients with a productive cough and fever with chest radiographs, AFB microscopy and, where affordable, routine GeneXpert MTB/RIF testing should be considered in local diagnostic protocols. In this population, high CRP was associated with TB diagnosis, requiring further investigation to establish its value as an additional diagnostic or screening tool. A high proportion of patients with diabetes and respiratory symptoms had TB (40%), emphasising the particular need for active screening in this patient group. Given 21% of patients in this study had had previous TB and thus are at increased risk of chronic lung damage, future treatment guidelines should also consider incorporating bronchiectasis management. The AMD-LFA identified a single (later culture confirmed) case of melioidosis and significantly reduced the time to diagnosis. It is a potentially useful test in patients at high risk of melioidosis, although the cost-effectiveness of instituting such a test routinely will depend on the local prevalence of melioidosis. Understanding the local burden of infections that are not susceptible to first-line empirical treatment in adults is essential for structuring syndromic pathways tailored to the population and may help to reduce inappropriate use of antimicrobials in the context of growing resistance.

## Data Availability

Data are available from the authors upon reasonable request and with permission of MORU data management committee.
